# Need to Focus on Occupational Health Issues

**DOI:** 10.4103/0970-0218.40888

**Published:** 2008-04

**Authors:** HT Pandve, PA Bhuyar

**Affiliations:** Department of Community Medicine, Dr. D.Y. Patil Medical College, Pimpri, Pune - 411 018, Maharashtra, India. E-mail: dr_harshalpandve@yahoo.co.in

Sir,

India is one of the largest and the most important developing countries of the world. In this country, public health emphasizes more on communicable diseases, malnutrition and reproductive healthcare. Majority of the population is working in industrial sector. Industrial revolution as well as globalization is increasing the burden of occupational hazards and changing occupational morbidity drastically. Still occupational health is seen as a secondary issue while formulating health policy and health-related programmes.

As per the Director General of Factory Advisory Services and Labour Institutes Report (1998), there were 300,000 registered industrial factories and more than 5000 chemical factories in India, employing over half a million workers. Approximately 8.8 million workers were employed in various factories.(1) In India, occupational health is more than simply a health issue, which includes child labour, poor industrial legislation, vast informal sector, less attention to industrial hygiene and poor surveillance data.(2) Statistics for the overall incidence and prevalence of occupational disease and injuries for the country is inadequate. The major occupational diseases morbidity of concern in India include silicosis, musculoskeletal injuries, coal workers' pneumoconiosis, chronic obstructive lung diseases, asbestosis, byssinosis, pesticide poisoning and noise-induced hearing loss.(2) According to Leigh *et al.*, the annual incidence of occupational disease was between 924,700 and 1,902,300, leading to over 121,000 deaths in India.(3) According to a survey of injury incidence in agriculture conducted in Northern India, an annual incidence of 17 million injuries per year (2 million moderate to serious events), and 53,000 deaths per year was estimated.(4) While India experiences an economic transition, occupational research approach should balance between understanding the modern industrial exposures and health risks of traditional sectors like agriculture and plantations. Lack of education, unawareness of hazards in one's occupation, general backwardness in sanitation, poor nutrition and climatic proneness to epidemics aggravate worker's health hazards in the work environment. Despite the existence of law that prohibits a paid work from children under the age of 14 years, an estimated 70-115 million children are part of the Indian workforce. Child labour in the agriculture sector accounts for 80% of child labourers in India and 70% of working children globally.(5)

In India, occupational health is not integrated with primary healthcare, and it is the mandate of the Ministry of Labour, not the Ministry of Health. Occupational health in India has to compete with primary health and curative health for its budget. In the context of legislations, the major legal provisions for the protection of health and safety at workplace are the Factories Act and Mines Act. The Factories Act, 1948, deals with occupational health and safety as well as welfare of workers employed in a factory. However, more than 90% of the Indian labour force does not work in factories; hence, they fall outside the purview of the Act.(1) A broad insight into the existing occupational health laws in India explicably brings out the verity of non-implementation of such laws, considering the present scenario with respect to the workers' health conditions.(6)

Occupational health not only deals with work-related disorders or diseases, but it also encompasses all factors that affect workers' health. With changing scenario, there is need to understand the risk factors of modern occupational hazards. India urgently requires modern occupational health safety (OHS) legislation with adequate enforcement machinery and establishment of centres of excellence in occupational medicine to catch up with the rest of the world.
